# Development and Biomechanics of *Grewia lasiocarpa* E. Mey. Ex Harv. Trichomes Exudate

**DOI:** 10.3390/plants12112198

**Published:** 2023-06-01

**Authors:** Nneka Augustina Akwu, Yougasphree Naidoo, Moganavelli Singh, Yaser Hassan Dewir, Katalin Magyar-Tábori, Makhotso Lekhooa, Adeyemi Oladapo Aremu

**Affiliations:** 1 Biology Cluster, School of Life Sciences, Westville Campus, University of KwaZulu-Natal, Private Bag X54001, Durban 4000, South Africa; naidooy1@ukzn.ac.za (Y.N.); singhm1@ukzn.ac.za (M.S.); oladapo.aremu@nwu.ac.za (A.O.A.); 2 Indigenous Knowledge Systems Centre, Faculty of Natural and Agricultural Sciences, North-West University, Private Bag X2046, Mmabatho 2790, South Africa; 3 Preclinical Drug Development Platform, Faculty of Health Sciences, North-West University, Private Bag X6001, Potchefstroom 2520, South Africa; makhotso.lekhooa@nwu.ac.za; 4 Plant Production Department, College of Food and Agriculture Sciences, King Saud University, Riyadh 11451, Saudi Arabia; ydewir@ksu.edu.sa; 5 Research Institute of Nyíregyháza, Institutes for Agricultural Research and Educational Farm (IAREF), University of Debrecen, P.O. Box 12, 4400 Nyíregyháza, Hungary; mtaborik@gmail.com

**Keywords:** Malvaceae, transmission electron microscopy, peltate, capitate, morphology, medicinal plant

## Abstract

*Grewia lasiocarpa* E. Mey. Ex Harv., Malvaceae (forest raisin) is a tropical small tree or shrub valued for its ecological importance as well as its nutritional, antioxidant, antibacterial, and anti-cancer properties as well as its ecological and ornamental importance. Glandular and non-glandular trichomes are present on the fruits, stem bark and leaves of *G. lasiocarpa* and these trichomes are the first line of defense. They are important structures that plants use to combat biotic and abiotic stress. The development of *G. lasiocarpa* trichomes and the biomechanics of the exudates present in the glandular (capitate) trichome were investigated for the first time using advanced microscopy techniques [Scanning electron microscope (SEM) and Transmission electron microscope (TEM)]. The pressurized cuticular striations may play a role in the exudates’ biomechanics, i.e., releasing secondary metabolites present in the capitate trichome, which was observed to be multidirectional. The presence of many glandular trichomes on a plant implies an increase in the amount of phytometabolites. A common precursor for the development of trichomes (non-glandular and glandular) was observed to be DNA synthesis associated with a periclinal cell division, thus the final fate of the cell is determined by cell cycle regulation, polarity, and expansion. The glandular trichomes of *G. lasiocarpa* are multicellular and polyglandular, while the non-glandular (glandless) trichomes are either single-celled or multicellular. Since, trichomes ‘house’ phytocompounds of medicinal, nutritional, and agronomical benefits; the molecular and genetic study of the glandular trichomes of *Grewia lasiocarpa* will be beneficial to humanity.

## 1. Introduction

The genus *Grewia* was named after Nehemiah Grew (1641–1712), an early plant anatomist and physiologist, species categorised under the genus *Grewia* L. are typically small trees, shrubs, climbers and lianas, and they are distributed worldwide [1], with the most frequent occurrence in Africa and Indo-Malayan regions [1,2]. *Grewia* spp. are over 400 distributed in Australia, Africa and Asia [2]. They are predominant in South Africa, Madagascar, Himalayan regions, India, Pakistan, China, Myanmar, Pacific islands (Tonga and Samoa), Malaysia, Thailand, northern Australia and Bangladesh, with a total of 690 published binomial [3,4]. In West Africa, Nigeria has the most abundant distribution of *Grewia* spp. [5].

*Grewia lasiocarpa* E. Mey. ex Harv. (Malvaceae) is a deciduous, fast-growing tropical shrub or small tree, that usually grows on marginal lands [2]. It has nutritive value, antioxidant, antibacterial, and anti-cancer properties [6,7]. Plants of this genus also have similar properties; *Grewia asiatica* [8], *Grewia optiva* [9], *Grewia tilii.folia* Vahl [10], *Grewia flava* DC. [11], *Grewia tembensis* (Fresen) [12], *Grewia serrulata* [13], and *Grewia hirsute* [14]. In addition to their medicinal values, plants of this genus, also have ecological, and ornamental importance [2]. Therefore, for their sustainable use and the maintenance of biodiversity, scientific study and conservation of these plants are important.

Every living organism has a means of protection from external biotic and abiotic factors, and for plants, trichomes are considered the first structures associated with protection from these factors [15]. There are two types of trichomes viz., glandular and non-glandular [16], the development of these trichomes could be genetically [17] or epigenetically controlled [18]. They occur in a wide range of sizes (length, breadth), colours, and numbers [19,20]. They constitute 1–3% of plant fresh weight and sometimes have high toxicity [21,22]. Trichomes are small, epidermal hair-like structures, that protect plants from external stressors, produce and store secondary metabolites. Trichomes shield plants from Ultra-Violent radiation, herbivores, and excessive water loss and they play an important role in plant adaptation. They are present on leaves, stembark, and fruits of *Grewia* species [1,23]. Different morphological variations occur in plant trichomes although certain degree of similarity may be observed in species of the same genus and families [24,25]. The trichomes of the genus *Grewia* are not exempted from this either, as they occur in diverse shapes and sizes such as stellate, multangulate-stellate, T-shaped, simple [19,23]. According to [26] plants have adapted varied storage techniques which depend on the type of secretion. This adaptation is responsible for the variety of trichome morphologies observed in plants. This correlates with our findings in [19] as there are different trichomes morphology in *Grewia lasiocarpa* which contain different phytometabolites. Although their distribution within a plant differs considerably [22,27], phytometabolites such as alkaloids, flavonoids, essential oils, and phenols are present in glandular trichomes [28,29]. This variation in trichome morphology and phytometabolites that are present in the glandular trichomes as a result of multiple evolutionary events [30]. The presence of non-glandular and glandular trichomes has been reported on the leaves and stem bark of *Grewia lasiocarpa* [19]. Particularly in the leaf trichomes of the model plant *Arabidopsis thaliana*, the molecular mechanism of unicellular trichome development has been intensively studied [31,32]. Numerous developmental and environmental variables influence plant trichome initiation and morphogenesis. Salicylic acid (SA), gibberellins (GA), Cytokinin (CTK), and Jasmonic acid (JA), are examples of phytohormones that have been demonstrated to influence trichome initiation and morphogenesis [20,33]. Additionally, research has demonstrated that the expression of several genes, including GLABRA1 (GL1) and TRANSPARENT TESTA GLABRA1 (TTG1), is essential for the growth of trichomes [34,35]. Understanding the complex interactions between these variables can help explain how trichome formation is regulated and how it might be used in biotechnology and agriculture.

Most of the metabolites stored or secreted from glandular trichomes are hydrophobic [24], whereas in the capitate trichome, nonvolatile (sticky resinous) metabolites such as certain diterpenoids or acylsugars are present [36]. The hydrophobic feature of the metabolites enables them to stick to the surface of insects, which might be advantageous for luring pollinators and for defence. Plant age and genotype influence the number, development, and level of maturity of trichomes morphogenesis [37,38]. The developmental processes of *Grewia lasiocarpa* trichomes and the way the metabolites are released in the glandular trichomes are not yet known. 

The present study is aimed to investigate the biomechanics of exudates present in the glandular trichomes, as well as the developmental processes of the non-glandular and glandular trichomes of *Grewia lasiocarpa* E. Mey. ex Harv.

## 2. Results

**Figure 1:**
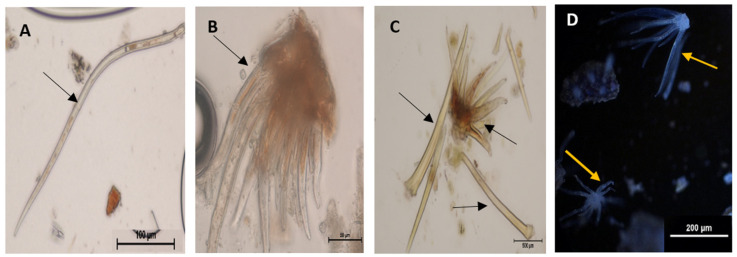
(**A**–**D**) Light micrographs (LM) of trichomes found on *Grewia lasiocarpa* E. Mey. Ex Harv. leaves* and stem bark powder. Non-glandular trichomes stained with Iodine (**A**,**B**), stained with potassium hydroxide (**C**), and stained with petroleum ether under ultra-violet light (D*).

**Figure 2:**
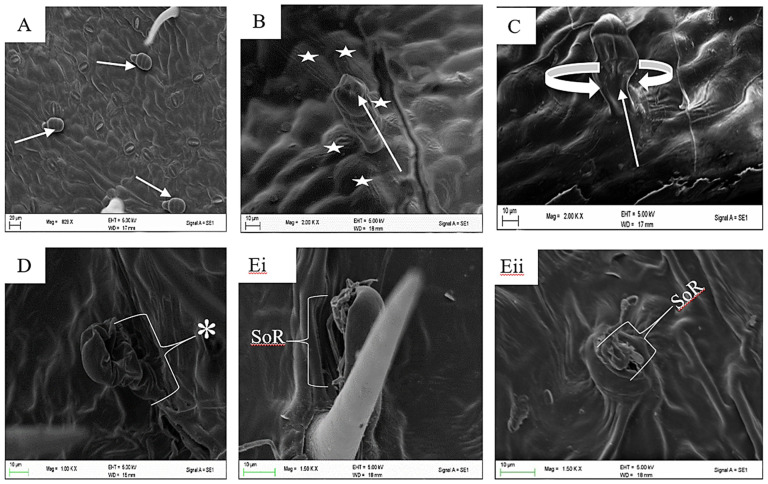
(**A**–**Eii**) The process of the release of metabolites in micrographs (**A**–**Eii**). Sequential rupture (broken cuticle) and release of metabolites in the subcuticular space of *Grewia lasiocarpa* E. Mey. Ex Harv. *Turgor pressure, capitate trichome (arrow), (SoR) Site of rupture (broken cuticle).

Trichomes are present on the leaves and stem bark of *Grewia lasiocarpa* E. Mey. Ex Harv. (Figure 1A–D), assisting in the plant’s overall coverage and defence. It was found that the constituents in the head of capitate trichomes are first released during the presecretory stage i.e., in the immature capitate trichome, that is not turgid due to the lack of metabolites (Figure 2A). The presecretory stage is followed by the secretory stage, which becomes evident by the appearance of a turgid subcuticular space, containing metabolites. This turgidity gives rise to an erect capitate trichome head (the star shapes indicate the striated cuticles in Figure 2B). The pressure created by the striated cuticles, moves upwards to the trichome head (Figure 2C), and consequently gives rise to an increase in the turgor pressure (asterisks) (Figure 2D). Thereafter, the metabolites are released out of the cuticle head which is hydrophobic [39]. The post-secretory stage is seen as a ruptured capitate trichome head (Figure 2Ei,Eii). The protective role of trichomes [40], is also evident in cuticular head (Figure 2Ei,Eii) as specialized structures that help secure the metabolites (non-polar and polar) before they are released. Despite the similar protective role shared by trichomes and cuticles the genetic linkages between cuticle formation and trichome development are yet to be understood [40]. The sequence of ruptured capitate head affirms the hypothesis of [20] that trichome senescence should occur from top to bottom.

**Figure 3:**
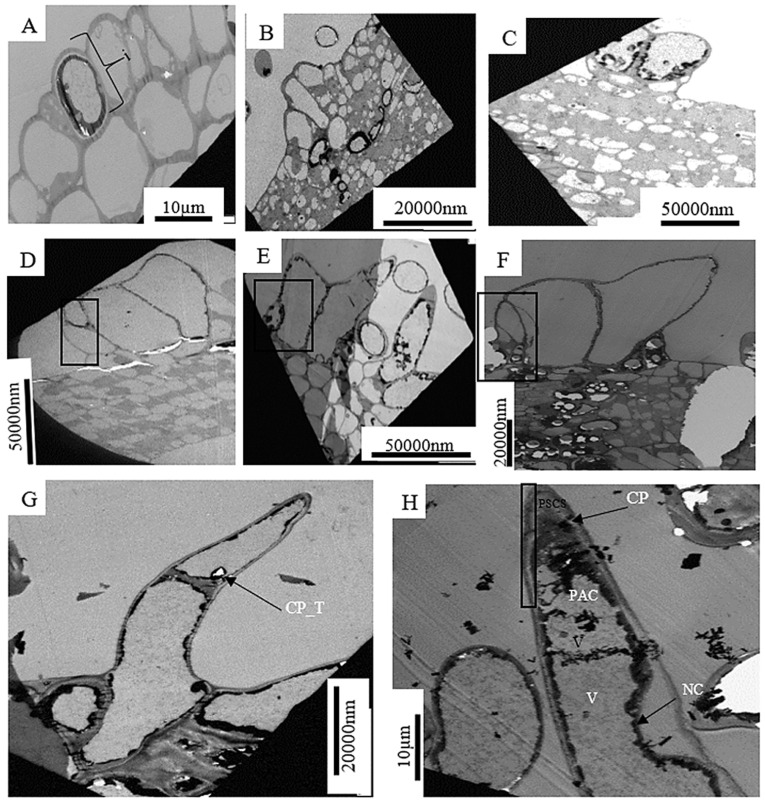
(**A**–**H**) Transverse Transmission electron microscopy sections of the successive stages of development of a simple structural trichome of *Grewia lasiocarpa* E. Mey. Ex Harv. The dark bands are those of the copper grids, thickened cell-wall can be seen in rectangular box. Arrow-trichome; Abbreviations: CP_T—calcium crystal; CP—calcium precipitate, PSCS—presumptive sub-cuticular space, PAC—presumptive apical cell, NC—neck cell, V—vacuole.

**Figure 4:**
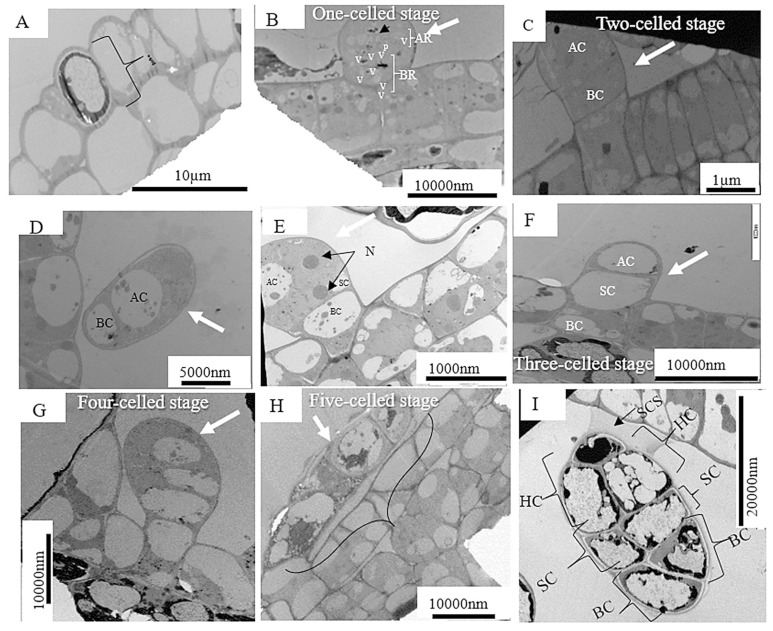
(**A**–**I**) Transmission electron micrographs of transverse sections during the successive stages of capitate trichome development of *Grewia lasiocarpa* E. Mey. Ex Harv. Abbreviations: AC—Apical cell, BC—basal cell, HC—head cell, SCS—sub-cuticular space.

**Figure 5:**
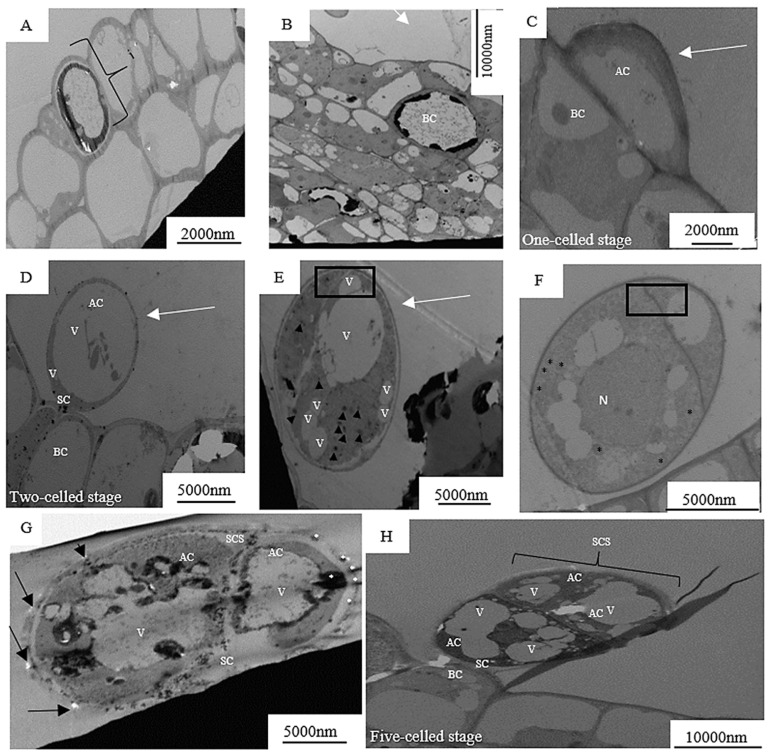
(**A**–**H**) Transmission electron micrographs of transverse section during successive stages of peltate trichome development of *Grewia lasiocarpa* E. Mey. Ex Harv. Abbreviations: AC—Apical cell, BC—basal cell, HC—head cell, SCS—sub-cuticular space, V—vacuole.

The transmission electron microscope (TEM), sections show the successive stages of trichomes development (Figure 3, Figure 4 and Figure 5). The first stage of trichome development involves deoxyribonucleic acid (DNA) synthesis (as in of all cellular divisions) in an actively dividing epidermal cell [initial cells] (Figure 3A). Then an anticlinal cell expansion occurs resulting in multicellular trichomes (Figure 3B). This is followed by nuclear migration and by the further divisions of certain cells (Figure 3D). Trichome branching then occurs with complete disintegration of the previously disintegrating cells (rectangular box) (Figure 3E). The maturation stages of trichomes begins (Figure 3F), which progresses (Figure 3G), and the presence of calcium crystals (CP_T) becomes evident by the tearing around the cells. Finally, the mature, simple trichome with precipitates of calcium (CP) around the cell wall can be seen in Figure 3H.

The development of the capitate trichome also starts with the epidermal cell. Figure 3A shows actively dividing initial cell (DNA synthesis), then the cell expands with the capitate trichome precursor which is composed of a single cell with a vacuolated basal region (BR) and an apical region (AR) containing the nucleus (arrow), several plastids and few vacuoles. Further cellular divisions occur which is the early stage of the three-celled stage. The early developmental stage of the capitate trichome after one periclinal division of the initial cell is indicated by the densely cytoplasmic apical cell (AC) and basal cell with few vacuoles (Figure 4C). This was also observed in fruit trichomes of cucumber, *Cucumis sativus* L. [20]. The AC retains its meristematic-like character but like the basal cells becomes less vacuolated (Figure 4D). Thereafter, nuclear migration occurs [nucleus (N)] with an early-stage formation of the stalk cell (SC). The formation of the SC becomes complete, giving rise to the three-cell stage (Figure 4F). Further cellular division occurs (Figure 4G) and an immature glandular trichome (GT) is formed in the presecretory stage (Figure 4H). Further cell division occurs (Figure 4G) and an immature glandular (GT) is formed in the presecretory stage. The immature glandular trichome becomes mature and enters the secretory stage (Figure 4I). The secretory stage has a short-stalked capitate trichome, with a thickened cell wall, sub-cuticular space (SCS), two head cells (HC), a narrow stalk cell (SC) and a basal cell (BC).

The developmental stages of a peltate trichome are shown in Figure 5A–H. Figure 5A presents an epidermal cell [i] showing actively dividing initial cell (DNA synthesis), and as similar with other trichome developmental processes, the cell expands forming the peltate trichome precursor which is a single cell with a vacuolated (initial) basal cell (Figure 5B). The early developmental stage of peltate trichome occurs after one periclinal division of initial cell giving rise to the apical cell and basal cell (Figure 5C). The apical cell with a large vacuole then expands, with a very narrow vacuolated stalk cell and the basal cell is also vacuolated (Figure 5D). Then the trichome is at the presecretory stage with two disk cells-(rectangular box), and more vacuoles (V) and enlarged plastids (P) (arrowhead) (Figure 5E). Nuclear migration occurs (Figure 5F), with a meristematic stage typical of an apical cell, lipid droplets (asterisks), fully developed cell wall between the two disk cells (rectangular box). More cellular divisions occur giving rise to sub-cuticular space, densely cytoplasmic apical cell, and stalk cell, (asterisks shows calcium precipitates, and arrows shows tear created by calcium crystals around the cell wall in Figure 5G). The peltate trichome matures showing the secretory stage, with more prominent layer of sub-cuticular space and highly vacuolated apical cell (Figure 5H).

## 3. Discussion

Trichomes or epidermal hairs are usually found in ferns and flowering plants and are used as a taxonomic tool [41]. They are usually found on fruits, stem, leaves and exterior or margins of sepals. Trichomes originate from epidermal cells [16], and the number of genes that control the development of trichomes is over 40 [42]. The number of cells (single-celled or multicellular trichome) in a mature trichome is determined by the number of formed, specialised epidermal cells [43,44]. Most of the non-glandular (glandless) trichomes are either single-celled or multicellular trichomes, while the glandular are usually multicellular and a further subdivision of single glandular or polyglandular [45]. Although the glandular trichomes of *Grewia lasiocarpa* are secretory due to the presence of metabolites; multicellularity is not a guarantee of the presence of phytometabolites as reported by [20,46,47]. Trichomes are a great model system for understanding cell polarity, cell cycle regulation, cell differentiation, as well as cell expansion [47,48]. Anatomical investigations of angiosperms, including studying of secretory structures such as trichomes (Figure 2A, Figure 3H, Figure 4I and Figure 5H), which exude discrete phytometabolites, are important for classification [49,50]. The origin, location, function, size, form, type of secretion and secreting ability have made it difficult to classify trichomes [43,51]. There are two broad classes of trichomes namely, glandular, and non-glandular (Figure 1, Figure 2, Figure 3 and Figure 4) [16]. Glandular trichomes have four distinct parts, namely, head cell (HC), neck cell (NC), stalk cell (SC) and foot cell (FC) [52], whereas non-glandular trichomes do not have these four distinct parts. 

Glandular and non-glandular trichomes are present on the leaves, sepals, fruits, and stem bark of *Grewia* spp. [4,53,54,55,56,57]. The glandular trichomes of species of this genus are composed of a multicellular head having either sessile or a short multicellular stalk, while four types of non-glandular trichomes with nine subdivisions are commonly found in plants of this genus. The presence of glandular and non-glandular trichomes on the leaves and stem bark of *Grewia lasiocarpa* has been previously reported [19]. Glandular trichomes contain substances of metabolic importance, that are released upon maturity [58,59]. In Figure 2A–E, it is evident that a form of force is needed to release the metabolites in the mature glandular trichomes as cuticular striations are absent around immature trichomes, but as the stages of development progress, cuticular striations are concentrated radially around the mature, erect capitate trichomes (Figure 2B), which means the head of the trichomes is under pressure. The cuticular striations were observed to still flank the capitate trichomes, but less pronounced (Figure 2C). It could be proposed that these striations are an indication of a constriction that is involved in the secretory process (Figure 2C). This finding suggests that the mature trichomes’ structural support and development are influenced by the cuticular striations. Additional research could look at the mechanics underlying this occurrence and its possible uses in biotechnology and plant breeding. The final process involves the release of the metabolites, which could be on any side of the trichome head (Figure 2Ei,Eii). This release of metabolites around the head is in agreement with the observation of [26]; who reported that cells at the tip of the trichome secrete substances directly onto the trichome’s surface.

It may be proposed that the presence of more glandular trichomes on a plant’s vegetative organs (stem, leaves, and roots) or reproductive organs (seeds or fruits) implies more phytometabolites will be obtained from that part of the plant [26,60,61,62]. The four distinct parts of glandular trichomes, namely head, neck, stalk, and foot cells were also observed to be present in the glandular trichomes on the leaves and stem bark of *Grewia lasiocarpa.* The glandular trichomes of *Grewia* spp. are composed of a multicellular head, having either sessile or a short multicellular stalk [56]. The secretory structures belonging to the Malvaceae have already been studied [63,64] including those of *Grewia flavescens* [65]. Figure 3, Figure 4 and Figure 5, support the assumption that trichomes originate from epidermal cells by periclinal division [16]. In addition, other cellular processes such as cell-cycle regulation, cell-death control, transcription, and machinery e.g., microtubule and actin cytoskeleton control the development of trichomes [32].

The stages of trichomes development observed and presented in Figure 3, Figure 4 and Figure 5: DNA synthesis evident in epidermal cell with biological activities (Figure 3 and Figure 5A), cell expansion, an outgrowth of epidermal cells (Figure 3 and Figure 5B), then a nuclear migration resulting in an increase of the epidermal cells (Figure 3C, Figure 4 and Figure 5F), followed by branching/partitioning (Figure 3E, Figure 4H and Figure 5E,F) and maturation (Figure 3 and Figure 5H), confirmed previously in reported findings [66,67]. According to these results, the biological processes of epidermal cells include branching/partitioning, maturation, nuclear migration, and cell expansion. This observation proposes that the genetic mechanisms may be responsible for the trichome branching and this characteristic may have evolved because of aberrant cell division patterns and gene regulation. Gaining insight into these underlying mechanics (pathways) may have significant effects on increasing crop yields and creating new plant-based products. 

Figure 3D–G, support the evolutionary origin of trichome branching including abnormal cell-plate formation model, which postulates that trichome branching occurs with a peculiar division pattern, controlled by genes responsible for branching [32]. These genes may be regulated by phytohormones. The pre-secretory stage of a capitate trichome in the leaf of *Grewia lasiocarpa* is evident in Figure 5E,F, with the appearance of two disk cells, large and numerous vacuoles. The cells in the head of a glandular trichome arise from the formation of cross walls as evident in Figure 4H and Figure 5G. Hence, the glandular trichomes of *Grewia lasiocarpa* are multicellular and polyglandular, while the non-glandular (glandless) trichomes are either single-celled or multicellular.

## 4. Materials and Methods

### 4.1. Plant Material

Fresh material of the leaves and stem bark of *Grewia lasiocarpa* E. Mey. ex Harv were collected from Umdoni Trust Park area of KwaZulu-Natal’s southern countryside, South Africa (30°23′13.28″ S, 30°40′17.11″ E), and a voucher material was deposited in the University of KwaZulu-Natal herbarium (Voucher number: Nneka02; identified by the curator, Dr. Syd Ramdhani).

### 4.2. Sample Preparation for Light Microscopy (LM) 

The dried leaves and stem bark of *Grewia lasiocarpa* E. Mey. Ex Harv were ground using a Waring blender (Christy and Norris—50158, England). A pinch of each was placed on clean glass slides and stained with 1 or 2 drops of iodine and potassium hydroxide solutions. A Nikon Eclipse E400 compound light microscope coupled to Nikon DS-Fi2 camera and image software-NIS-Elements was used to view the mixtures under bright field and under bright field and ultraviolet-2A (ex 330/380) illumination. 

### 4.3. Sample Preparation for Scanning Electron Microscopy (SEM)

The fresh plant material samples (leaves and stem bark) were cut into ca. 2–3 mm in lengths, and then fixed in 2.5% glutaraldehyde in 0.1 M phosphate buffer (pH 7.2) for 24 h at 4 °C. Then they were washed thrice for five min per wash and re-fixed in 0.5% Osmium tetroxide (OsO_4_) for 24 h at 4 °C. Thereafter, the plant material was rinsed thrice again for five min per wash, thereafter, dehydrated using graded series of ethanol (25%, 50%, 75%), twice for five min and a final 100% ethanol dehydration, twice for 10 min. A critical point drying process was carried out in Hitachi Critical Point Drier (CPD) (Hitachi, LTD. Tokyo, Japan). The samples were then placed on brass stubs on which a double-sided sticky adhesive carbon tape was initially stuck on to escalate conductivity between the plant material and the stage, and then the samples were coated wi.th gold using an automated Polaron SC 500 Module sputter coater (vacuum of 0.1 Torr for 2.5 min) sputter coater for 10 min. The topography of the plant material was then observed using a LEO 1450 SEM at an acceleration voltage of 5 kV and all the representative features examined were captured digitally using (computer program) NIS-D image software.

### 4.4. Sample Preparation for Transmission Electron Microscopy (TEM)

The fresh leaves of *Grewia lasiocarpa* were cut into 1–2 mm in lengths, and fixed with 2.5% glutaraldehyde in 0.075 M phosphate buffer (pH 7.4), at 4 °C for 24 h [68]. Then washed thrice (five min per wash) in 0.075 M phosphate buffer (pH 7.4). The leaves were thereafter, post-fixed for 1 h in 0.5% buffered osmium tetroxide (OsO_4_), pH 7.4 [69]. Then, washed again thrice with the phosphate buffer at an interval of five min per wash and dehydrated in a graded acetone series (30%, 50%, 75%, 100%). The acetone in the samples was steadily replaced by infiltration for 4 h using equal parts of low- viscosity 100% Spurr’s resin (Epon 812 recipe) and acetone, then embedded for 24 h using whole resin [70]. The samples were finally embedded in freshly prepared whole (Epon 812) resin, using silicone moulds and polymerized at 70 °C for 8 h in an oven. Ultra-thin sections of 0.5–2.0 µm were cut with a glass knife mounted on a Reichert Jung Ultracut-E ultramicrotome. The sections were picked with 100 mesh copper grids. The dried sections were thereafter post-stained with 2.5% uranyl acetate [71] and subsequently with 2.5% lead citrate solutions [72]. The sections were examined, and images captured with a Jeol 1010 electron transmission microscope at 100 kV accelerating voltage equipped with an Olympus Mega View III CCD (Soft imaging system GmBH, Münster, Germany).

## 5. Conclusions

In ferns and flowering plants, trichomes (epidermal hairs) are important taxonomic tools, and it is widely known that they can be found on a variety of plant parts, including fruits, leaves, stems and sepals. Over 40 genes are coordinated during the intricate formation of trichomes, which start as specialised epidermal cells. They come in various forms, both glandular and non-glandular, which means it can be difficult to classify them because of differences in of forms, both glandular and non-glandular, which means it can be difficult to classify them because of differences in their origin, function, location, form, size, type of secretion, and secretory characteristics. On their leaves, sepals, fruits, and stem bark, *Grewia* spp. plants have glandular and non-glandular trichomes, with the glandular trichomes made of multicellular components. The trichomes of *Grewia lasiocarpa* E. Mey. ex Harv. can be used as a taxonomic tool (classification and identification purposes) and they are proposed to be important for species-specific adaptation and ecological interactions. The transmission electron microscopy revealed the successive stages of *Grewia lasiocarpa* trichomes developmental processes, which includes DNA synthesis, cell expansion, nuclear migration, branching/partitioning, and maturation. The organogenesis and development of the *Grewia lasiocarpa* trichomes are different. The glandular, which as four distinct parts: head cell (HC), neck cell (NC), stalk cell (SC) and foot cell (FC)) and non-glandular (lacks HC, NC, SC, and FC), trichomes of *Grewia lasiocarpa* are metabolically active, but exudates are present only in the glandular (capitate) trichome. In the mature capitate trichomes, a turgid subcuticular space is observed; metabolites are released through the hydrophobic cuticle during the secretory stage after going through the pre-secretory stage. The presence of several glandular trichomes on a plant may indicate that there are more phytometabolites present. Trichomes play a protective role, as seen in the cuticular head, which aids in metabolite storage prior to their release. The cuticular striations observed around mature trichomes further demonstrates the role that trichomes may have in the synthesis and secretion of phytometabolites. Cuticular striations may appear on any side of the trichome head and help to release metabolites from mature glandular trichomes. During the secretory stage, the peltate trichomes also go through a similar developmental process, developing prominent subcuticular spaces and significantly vacuolated apical cells. The non-glandular trichomes are typically known to be for defense, but it is evident that they are metabolically active and thus their function is beyond structural. The process of trichome formation is regulated by certain yet unknown genes. The final fate of the non-glandular and glandular trichomes of *Grewia lasiocarpa* are determined by cell cycle regulation, polarity, and expansion. It is recommended that the genes that regulate (positively or negatively) the development of these trichomes which may be associated with phytohormones (environmental cues) should be identified and characterised. This would give more insights to the ecological and evolutionary implications of trichomes of this genus.

## Data Availability

Data are available from the authors upon request.
